# Development of a risk estimation model for condomless sex among college students in Zhuhai, China: a cross-sectional study

**DOI:** 10.1186/s12889-024-18183-9

**Published:** 2024-03-08

**Authors:** Ying Huang, Yi Zhou, Yeting Hong, Wencan Dai, Kaihao Lin, Yawei Liu, Yao Yan, Shanzi Huang, Xiaofeng Li, Yi Yang, Hongbo Jiang

**Affiliations:** 1 Department of Epidemiology and Biostatistics, School of Public Health, Guangdong Pharmaceutical University, Guangzhou, China; 2 Zhuhai Center for Disease Control and Prevention, Zhuhai, China; 3 Institute for Global Health, University College London, London, UK

**Keywords:** College students, Condomless sex, Condom use, LASSO, Nomogram

## Abstract

**Background:**

Condom use at last intercourse is an effective indicator for human immunodeficiency virus (HIV) prevention. To identify at-risk individuals and improve prevention strategies, this study explored factors associated with condomless sex at last intercourse in the last year and developed a risk estimation model to calculate the individual possibility of condomless sex among college students in Zhuhai, China.

**Methods:**

A cross-sectional study was conducted among 1430 college students who had sex in the last year from six universities in Zhuhai. The least absolute shrinkage and selection operator (LASSO) and logistic regression were performed to explore the predictors of condomless sex. The nomogram was constructed to calculate the individual possibility of condomless sex. Discrimination and calibration of the nomogram were evaluated using the area under the receiver-operator characteristic curve (AUROC) and the calibration curve.

**Results:**

The proportion of students who had condomless sex at last intercourse was 18.2% (260/1430). Students who had experienced more types of intimate partner violence (aOR, 1.58; 95% CI, 1.31 ~ 1.92) and had anal sex (aOR, 1.75; 95% CI, 1.06 ~ 2.84) were more likely to have condomless sex. Students who had heterosexual intercourse (aOR, 0.37; 95% CI, 0.21 ~ 0.70), used condoms at first sex (aOR, 0.20; 95% CI, 0.14 ~ 0.27), had high attitudes towards condom use (aOR, 0.87; 95% CI, 0.80 ~ 0.95) and self-efficacy for condom use (aOR, 0.84; 95% CI, 0.78 ~ 0.90) were less likely to have condomless sex. The nomogram had high accuracy with an AUROC of 0.83 and good discrimination.

**Conclusions:**

Intimate partner violence, anal sex, condom use at first sex, attitude towards condom use, and self-efficacy for condom use were associated with condomless sex among college students. The nomogram was an effective and convenient tool for calculating the individualized possibility of condomless sex among college students. It could help to identify individuals at risk and help universities and colleges to formulate appropriate individualized interventions and sexual health education programs.

**Supplementary Information:**

The online version contains supplementary material available at 10.1186/s12889-024-18183-9.

## Introduction

Condom use is effective in preventing the transmission of human immunodeficiency virus (HIV) and sexually transmitted infections (STIs) [1]. However, the proportion of condomless sex among college students remains high [2]. Currently, condomless sexual transmission remains the main route of HIV transmission among college students [3]. The number of newly diagnosed people living with HIV among college students in China was increasing at a rate of 30–50% per year [4]. In addition, a survey of 35,383 unmarried female college students in China found that the proportion of unintended pregnancy was 31.8%, of which 53.5% experienced two or more pregnancies [5]. The current situation has reminded us that condomless sex among college students is still an issue that deserves attention. With greater self-control and adaptability, college students had the opportunity to change their behavior. Therefore, understanding factors associated with condomless sex at this stage could help college students to break their risky sexual behavior habits in time.

Condom use at the last intercourse is one of the most common measures used to assess condom use [6]. A large number of studies have shown that condomless sex is influenced by a wide range of factors, including social demographic characteristics, behaviors, substance use, psychological factors and so on [7–11]. For example, condom use at the first sex [12–14], experience of intimate partner violence (IPV) [8, 15], and self-efficacy of condom use [10, 16, 17] were significant predictors of condomless sex. Previous studies have usually used logistic regression to access the risk factors for condomless sex, but the effect of different risk factors varies. When many factors coexist, it is important to identify the salient factors and to help individuals recognize or identify their risks based on these factors. Identifying students at risk of condomless sex not only helps to prevent condomless sex at an early stage, but also protects their sexual health. The nomogram is a graphical tool based on a regression model, which can distinguish the differences between each factor [18]. In the nomogram, each factor corresponds to different numerical points, which could be calculated to obtain the total score of each factor for each individual in relation to the risk of the event, reflecting their personal risk. Nowadays, the nomogram has been widely used to predict a variety of clinical outcomes [19–23], which formed the basis for the formulating treatment cases and patient management. However, it has rarely been applied school health settings. The nomogram can quantify the risk of individual events without the need for complex formula calculations, which is easily accepted by health educators and facilitates better health education. Therefore, a cross-sectional survey was conducted to explore the significant predictors of condomless sex, and then nomogram to predict the individual possibility of condomless sex at last intercourse was developed and validated among college students in Zhuhai, China.

## Methods

### Population

A cross-sectional survey was conducted among college students from six out of the seven universities in Zhuhai City, China. Inclusion criteria for participants included 1) college students enrolled in six universities in Zhuhai; 2) those who consented to participate in the survey and completed the questionnaire, and minors who had consent from their guardians; 3) self-reported history of sexual intercourse (including vaginal, anal, or oral sex).

Exclusion criteria for participants included 1) those with mental illness and intellectual disability; 2) those who did not fully understand the informed consent process and did not consent to the questionnaire, and minors who had no consent from their guardians; 3) those who self-reported no sexual experience.

### Study setting

The sample size estimation formula for a cross-sectional study was used to calculate the required sample size. With a proportion of condomless sex at last intercourse among college students in Zhuhai (P) of 7.3%, a precision error (d) of 0.1P, and a confidence level of 95%, the required sample size was calculated to be 5366, considering a nonresponse rate of 10% [24]. A multistage sampling method was used to recruit college students in Zhuhai City from September to October 2019. In the first stage, probability proportional to size sampling was used to determine the sample size of each university, where the probability of selecting a student was proportional to the total number of students in each university, ensuring the representativeness of the sample and improve the accuracy of the study results [25]. In the second stage, all departments/colleges in each university were classified into one of the four fields of major (literature, science, arts and medicine). One or two specific majors were then randomly selected from each field of major. In the third stage, random cluster sampling was used to select between one and five classes from each grade of the specific majors. All students in the selected classes were invited to self-administer the online questionnaire via a link. Using the sampling method described above, a total of 12,235 students were recruited, and 1430 students who had sex in the last year were included in this study. Supplementary Fig. 1 shows the schematic presentation of sampling procedure for this study. The study was approved by the Ethics Committee of the Zhuhai Centre for Disease Control and Prevention.

### Data collection

Data were collected using an electronic questionnaire. The survey was organized and coordinated by the Department of Epidemiology and Biostatistics, School of Public Health, Guangdong Pharmaceutical University. The questionnaire investigators received uniform training. Senior investigators examined the collected questionnaires for quality control to ensure the accuracy of the data.

### Variables

The self-designed electronic questionnaire was developed based on the standardized National AIDS Sentinel Surveillance Questionnaire, specifically designed for college students [26], and the existing literature [6, 27–31]. The questionnaire collected the following data: 1) Sociodemographic characteristics included sex, age, the field of major, grade, residence, monthly disposable income, and resident student. 2) Behavioral characteristics included sexual orientation, sexual partner seeking, ever having had vaginal sex, ever having had oral sex, ever having had anal sex, ever having had heterosexual intercourse, age at sexual debut (**<** 18 years, ≥18 years), condom use at first sex, experience of IPV [32, 33], the number of types of IPV experienced, ever having been tested for HIV, awareness of HIV-related knowledge and willingness to receive HIV-related education. IPV was measured with four items: ① Do you have an intimate partner (boyfriend/girlfriend, spouse, or other sexual partners)? ② Have you ever been threatened with violence, treated with violence (e.g. slapping, hitting, kicking, pushing, throwing things at you) or fought with your intimate partners? ③ Have your intimate partners ever insisted on having sex with you or force you to have sex when you don’t want to? ④ Have your intimate partners ever verbally threatened, demeaned in front of others, ridiculed for your appearance, forced to get high or drunk, or stalked, or having property destroyed or damaged? [32, 33]. The types of IPV experienced were physical violence, sexual violence, and psychological violence. There were five items ‘no intimate partner, no experience, one type, two types, three types’. 3) Substance use before sex included smoking before sex, using e-cigarettes before sex, drinking alcohol before sex, and using club drugs before sex. 4) Psychosocial factors included attitudes, norms, and self-efficacy for condom use which were measured by the Sexual Risk Behavior Beliefs and Self-efficacy (SRBBS) scale [28] with a Cronbach’s α of 0.943. A 10-item short version of the Big Five Inventory [29], Form V of the Sensation Seeking Scale for adolescents [30], and the 10-item Sexual Compulsivity Scale [31] were used to measure the five-factor model of personality, sensation seeking, and sexual compulsivity, and their internal consistency estimates (i.e., Cronbach’s α) were 0.652, 0.857, and 0.941, respectively. Responses to the SRBBS and sexual compulsivity were given on four-point scales labeled ‘1’ (strongly disagree), ‘2’ (disagree), ‘3’ (agree), and ‘4’ (strongly agree). Responses to the five-factor model of personality and sensation seeking were given on five-point scales labeled ‘1’ (strongly disagree), ‘2’ (disagree), ‘3’ (unknow), ‘4’ (agree), and ‘5’ (strongly agree). 5) Condomless sex, which was the outcome variable in this study, was defined as not using a condom during the last sexual intercourse in the last year [6].

### Statistical analysis

In the univariate analysis, non-normal continuous variables are expressed as the median (M) and interquartile range (IQR) and were compared using the Mann-Whitney test. Categorical variables were compared using the χ^2^ test.

The dataset was randomly split into a derivation cohort (70%) and a validation cohort (30%). We obtained all variables from the questionnaires and used the least absolute shrinkage and selection operator (LASSO) regression to select potential variables associated with condomless sex with 10-fold cross-validation. LASSO regression can be used to screen variables and adjust for complexity while fitting a generalized linear model. It eliminates the weaker factors with greater penalties, whose coefficient shrinks towards zero, and keeps the most vital factors in the model [20, 23]. Stepwise multivariable logistic regression analysis was then used to determine the final independent predictors of condomless sex to construct the nomogram [23, 34]. The nomogram proportionally converts each regression coefficient in the multivariable logistic regression into a score. Finally, the total score for each participant was used to calculate the predicted probability of condomless sex by functional transformation [19]. We used 1000 bootstrap resamples as internal validation to estimate the accuracy of the model. The area under the receiver-operator characteristic curve (AUROC) and the calibration curves were used to evaluate the discrimination and calibration of the model, respectively. All analyses were performed using R, version 4.0, and *P* < 0.05 was considered statistically significant.

## Results

### Characteristics of college students

A total of 1430 students from six universities in Zhuhai who had sex in the last year were included in this analysis, of whom 18.2% (260/1430) had condomless sex at last intercourse. As shown in Table 1, there were 57.6% males and 42.4% females with a mean age of 20.98 (1.43) years. Most of them had an urban residence (73.1%), had a disposable income of more than 2000 yuan per month (73.6%) and lived in school dormitories (96.8%) (Table 1). As shown in Table 2, the most of the students identified themselves as heterosexual (85.5%) and had had heterosexual intercourse (94.6%). The proportions who had ever had vaginal sex, oral sex and anal sex were 91.3%, 64.7% and 8.9%, respectively. Approximately one in five (20.3%) students had their first sexual intercourse before the age of 18, of whom 23.4% did not use a condom the first time they had sex. The proportion of students who had ever experienced IPV was 18.7%. Only 7.6% of the students had been tested for HIV, and 83.4% had acquired HIV-related knowledge. However, 6.4% of students were reluctant to accept HIV-related education (Table 2). As shown in Table 3, The proportions of smoking, using e-cigarettes, drinking alcohol and using club drugs before sex were 24.3%, 8.4%, 35.1% and 2.1%, respectively. The median scores for attitude towards condom use, condom use norms, and condom use self-efficacy were 15.0 (IQR, 12.0–15.0), 15.0 (IQR, 12.0–15.0), and 14.0 (IQR, 11.0–15.0), respectively. The median score for sexual compulsivity was 18.0 (IQR, 11.0 ~ 21.0) (Table 3).

**Table 1:** Sociodemographic characteristic among college students who had sex in the last year

Variables	Total (*N* = 1430)	Condom use group (*n* = 1170)	Condomless sex group(*n* = 260)	***χ*** ^*2*^	*P* Value
Sex				1.043	0.307
Male	823(57.6)	666(56.9)	157(60.4)		
Female	607(42.4)	504(43.1)	103(39.6)		
Age, mean (SD^a^), y	20.98(1.43)	20.96(1.33)	21.08(1.79)	1.167	0.244
Field of major				2.128	< 0.001
Literature	584(40.8)	483(41.3)	101(38.8)		
Science	452(31.6)	369(31.5)	83(31.9)		
Art	364(25.5)	297(25.4)	67(25.8)		
Medicine	21(1.5)	19(1.6)	2(0.8)		
N/A	9(0.6)	2(0.2)	7(2.7)		
Grade				0.779	0.712
Freshmen	233(16.3)	188(16.1)	45(17.3)		
Sophomore	410(28.7)	341(29.1)	69(26.5)		
Junior	508(35.5)	414(35.4)	94(36.2)		
Senior	279(19.5)	227(19.4)	52(20.0)		
Residence				0.016	0.899
Urban	1046(73.1)	855(73.1)	191(73.5)		
Rural	384(26.9)	315(26.9)	69(26.5)		
Monthly disposable income, yuan				2.764	0.251
0~	377(26.4)	301(25.7)	76(29.2)		
2001~	868(60.7)	722(61.7)	146(56.2)		
≥4000	185(12.9)	147(12.6)	38(14.6)		
Resident student				1.997	0.158
Yes	1384(96.8)	1136(97.1)	248(95.4)		
No	46(3.2)	34(2.9)	12(4.6)		

^a^SD referred to standard deviation

**Table 2:** Behavioral characteristics among college students who had sex in the last year

Variables	Total (*N* = 1430)	Condom-using group (n = 1170)	Condomless sex group (n = 260)	***χ*** ^*2*^	*P* Value
Sexual orientation				9.903	0.002
Heterosexual	1222(85.5)	1016(86.8)	206(79.2)		
Non-heterosexual	208(14.5)	154(13.2)	54(20.8)		
Seeking sexual partners				9.120	0.010
Internet	416(29.1)	333(28.5)	83(31.9)		
Non-Internet	579(40.5)	495(42.3)	84(32.3)		
Both	435(30.4)	342(29.2)	93(35.8)		
Ever having vaginal sex				15.604	< 0.001
Yes	1305(91.3)	1084(92.6)	221(85.0)		
No	125(8.7)	86(7.4)	39(15.0)		
Ever having oral sex				9.796	0.002
Yes	925(64.7)	735(62.8)	190(73.1)		
No	505(35.3)	435(37.2)	70(26.9)		
Ever having anal sex				42.063	< 0.001
Yes	127(8.9)	77(6.6)	50(19.2)		
No	1303(91.1)	1093(93.4)	210(80.8)		
Ever having heterosexual intercourse				11.165	0.001
Yes	1353(94.6)	1118(95.6)	235(90.4)		
No	77(5.4)	52(4.4)	25(9.6)		
Age at sexual debut				36.176	< 0.001
< 18	290(20.3)	202(17.3)	88(33.8)		
≥18	11,140(79.7)	968(82.7)	172(66.2)		
Condom use at first sex				198.763	< 0.001
Yes	1095(76.6)	983(84.0)	112(43.1)		
No	335(23.4)	187(16.0)	148(56.9)		
The number of types of intimate partner violence experienced ^a^				62.016	< 0.001
No intimate partner	48(3.4)	35(3.0)	13(5.0)		
No experience	1114(77.9)	946(80.9)	168(64.6)		
One	177(12.4)	141(12.1)	36(13.8)		
Two	42(2.9)	22(1.8)	20(7.7)		
Three	49(3.4)	26(2.2)	23(8.9)		
Ever having HIV testing				1.793	0.196
Yes	109(7.6)	84(7.2)	25(9.6)		
No	1321(92.4)	1086(92.8)	235(90.4)		
Awareness of HIV-related knowledge				3.338	0.068
Yes	1193(83.4)	986(84.3)	207(79.6)		
No	237(16.6)	184(15.7)	53(20.4)		
Willingness to receive HIV-related education				9.923	0.002
Yes	1338(93.6)	1106(94.5)	232(89.2)		
No	92(6.4)	64(5.5)	28(10.8)		

^a^Types of intimate partner violence experienced included physical, verbal, and sexual types

**Table 3:** Substance use and psychosocial characteristics among college students who had sex in the last year

Variables	Total (N = 1430)	Condom-using group (n = 1170)	Condomless sex group(n = 260)	***χ*** ^*2*^	*P* Value
Substance use before sex					
Smoking before sex				9.146	0.002
Yes	347(24.3)	265(22.6)	82(31.5)		
No	1083(75.7)	905(77.4)	178(68.5)		
Using e-cigarettes before sex				16.012	<0.001
Yes	120(8.4)	82(7.0)	38(14.6)		
No	1310(91.6)	1088(93.0)	222(85.4)		
Drinking alcohol before sex				18.235	<0.001
Yes	502(35.1)	381(32.6)	121(46.5)		
No	928(64.9)	789(67.4)	139(53.5)		
Using club drugs before sex				25.452	<0.001
Yes	30(2.1)	14(1.2)	16(6.2)		
No	1400(97.9)	1156(98.8)	244(93.8)		
Psychosocial characteristics					
Score on SRBBS, M (IQR)^a^					
Attitudes towards condom use points	15.0 (12.0 ~ 15.0)	15.0 (13.0 ~ 15.0)	12.0 (11.0 ~ 15.0)	0.294	< 0.001
Norms on condom use points	15.0 (12.0 ~ 15.0)	15.0 (12.0 ~ 15.0)	12.0 (10.0 ~ 15.0)	0.215	< 0.001
Self-efficacy of condom use points	14.0 (11.0 ~ 15.0)	15.0 (12.0 ~ 15.0)	11.0 (9.0 ~ 12.0)	0.319	< 0.001
Five-factor model of Personality, M (IQR)					
Extraversion	6.0(6.0 ~ 7.0)	6.0(6.0 ~ 7.0)	6.0(6.0 ~ 7.8)	0.021	0.419
Agreeableness	6.0(6.0 ~ 7.0)	6.0(6.0 ~ 7.0)	6.0(5.0 ~7.0)	0.029	0.269
Conscientiousness	6.0(6.0 ~ 7.0)	6.0(6.0 ~ 7.0)	6.0(6.0 ~ 8.0)	0.012	0.643
Neuroticism	6.0(5.0 ~ 7.0)	6.0(5.0 ~ 7.0)	6.0(5.0 ~ 7.0)	0.016	0.535
Openness	8.0(6.0 ~ 9.0)	8.0(6.0 ~ 9.0)	8.0(6.0 ~ 9.0)	0.033	0.214
Sensation seeking, M (IQR)					
Experience seeking	6.0(4.0 ~ 7.0)	6.0(4.0 ~ 7.0)	6.0(4.0 ~ 7.0)	0.025	0.337
Boredom susceptibility	6.0(5.0 ~ 7.0)	6.0(5.0 ~ 7.0)	6.0(5.0 ~ 7.0)	0.010	0.708
Thrill adventure seeking	6.0(5.0 ~ 8.0)	6.0(5.0 ~ 8.0)	6.0(5.0 ~ 8.0)	0.020	0.439
Disinhibition	4.0(3.0 ~ 6.0)	4.0(3.0 ~ 5.0)	4.0(3.0 ~ 6.0)	0.089	0.001
Sexual compulsivity	18.0 (11.0 ~ 21.0)	17.0 (10.0 ~ 21.0)	20.0 (13.0 ~ 23.0)	0.151	< 0.001

^a^SRBBS referred to sexual risk behavior beliefs and self-efficacy. M referred to median. IQR referred to interquartile range

Compared to the condom-using group, the condomless group had higher proportions of students who were non-heterosexual (*P* = 0.002), had oral (*P* = 0.002) and anal sex (*P* < 0.001), had the first sex before the age of 18 (*P* < 0.001), did not use condoms at first sex (*P* < 0.001), and had experienced more than two types of IPV (*P* < 0.001) (Table 2). In addition, the condomless group was more likely to smoke (*P = *0.002), use e-cigarettes (*P* < 0.001), drink alcohol (*P* < 0.001) and use club drugs (*P* < 0.001) before sex than the condom-using group (Table 3), while the condomless group was less likely to have vaginal sex (*P* < 0.001), have heterosexual sex (*P* = 0.001) and be willing to receive HIV-related education (*P* = 0.002) than in the condom-using group (Table 2). In terms of scores, attitude towards condom use (*P* < 0.001), condom use norms (*P* < 0.001), and condom use self-efficacy (*P* < 0.001) were lower in the condomless group than in the condom-using group, while sexual compulsivity (*P* < 0.001) was higher in the condomless group than in the condom-using group (Table 3).

### Development and validation of a nomogram for Condomless sex

All the potential factors associated with condomless sex were included in the LASSO regression. After selection by LASSO regression (Supplementary Fig. 2), eight variables were retained, including ever having vaginal sex, ever having anal sex, having heterosexual intercourse, condom use at first sex, more types of IPV, attitudes towards condom use points, condom use self-efficacy points and sexual compulsivity points. After multivariable analysis, six variables remained that were independently statistically significant predictors of condomless sex. As shown in Table, students who had experienced more types of IPV (aOR, 1.58; 95% CI, 1.31 ~ 1.92) and had anal sex (aOR, 1.75; 95% CI, 1.06 ~ 2.84) were more likely to have condomless sex. However, students who had heterosexual intercourse (aOR, 0.37; 95% CI, 0.21 ~ 0.70), used condoms at first sex (aOR, 0.20; 95% CI, 0.14 ~ 0.27), had high scores on attitudes towards condom use (aOR, 0.87; 95% CI, 0.80 ~ 0.95) and self-efficacy for condom use (aOR, 0.84; 95% CI, 0.78 ~ 0.90) were less likely to have condomless sex (Table 4).

**Table 4:** Multivariable Logistic Regression Analysis of Predicting Condomless Sex

Variable	β^a^	*OR* (95% *CI*)	*P* Value
**Having heterosexual intercourse**, yes	−0.98	0.37(0.21 ~ 0.70)	0.002
**More types of intimate partner violence experienced** ^b^	0.46	1.58(1.31 ~ 1.92)	< 0.001
**Ever having anal sex**, yes	0.56	1.75(1.06 ~ 2.84)	0.027
**Condom use at first sex,** yes	−1.65	0.20(0.14 ~ 0.27)	< 0.001
**Attitudes towards condom use points**	−0.14	0.87(0.80 ~ 0.95)	0.001
**Condom use self-efficacy points**	−0.18	0.84(0.78 ~ 0.90)	< 0.001

^a^Unstandardized β coefficients were calculated from the multivariable logistic regression model

^b^Types of intimate partner violence experienced included physical, verbal, and sexual types

**Fig. 1:**
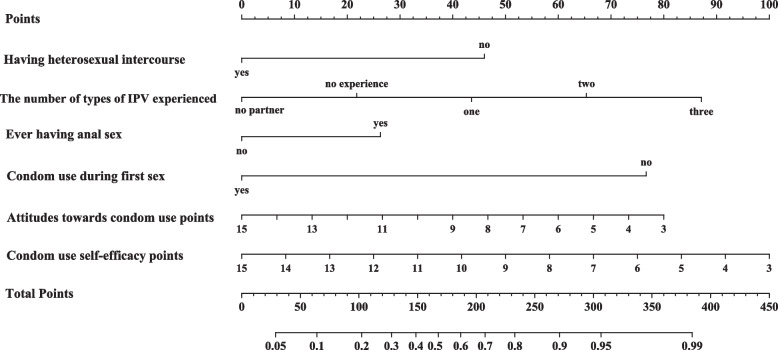
Nomogram for Predicting the Possibility of Condomless Sex and Its Predictive Performance. Nomogram for Predicting the Possibility of Condomless Sex in College Students Who Had Sex in the Last Year in Zhuhai, China. In the nomogram above, the options for each variable correspond to a particular point on the top row. The points for each variable are added together to give a total score, which refers to the probability on the bottom row. *IPV. Imitate partner violence

These independent predictors were used to construct a nomogram to estimate the individualized risk of condomless sex **(**Fig. 1**)**. In the nomogram, the options for each variable correspond to a particular point on the top row. The points of each variable are added together to give a total point, which corresponds to the probability on the bottom row. In addition, we have provided a list of the specific total point and corresponding probability of condomless sex in Supplementary Table 1. Based on the specific total point and the corresponding probability of condomless sex, we divided the college students into three subgroups. The possibility of condomless sex was less than 5% in the low-risk group (total points < 30), between 5 and 50% in the moderate-risk group (30 ≤ total points ≤ 170), and more than 50% in the high-risk group (total points > 170). We also compared the actual proportion of condomless sex with the predicted possibility in the three classified subgroups (Table 5). The proportion of condomless sex was 2.6% in the low-risk group which accounted for 29.9% of all students. The proportion of condomless sex was 19.1% in the moderate-risk group which accounted for 60.9% of all students. The proportion of condomless sex was 63.4% in the high-risk group which accounted for 9.2% of all students. The actual possibility of condomless sex differed significantly (*P* < 0.001) between the three subgroups.

**Table 5:** The association between different risk groups and actual outcome^a^

Risk category	Outcome		Overall
	Condom-using sex	Condomless sex	
Low risk	417(97.4)	11(2.6)	428(29.9)
Moderate risk	705(80.9)	166(19.1)	871(60.9)
High risk	48(36.6)	83(63.4)	131(9.2)
Overall	1170(81.8)	260(18.2)	1430 (100.0)

^a^Values are numbers (percentages) unless stated otherwise. Fisher exact probability test was applied. Bonferroni correction was used for pairwise comparisons and significant threshold was corrected as 0.05/3 = 0.017. Denoting: P-value of A vs B represents comparing the proportion of outcome between A and B

*P*-value among three group is less than 0.001

*P*-value of Low-risk group vs Moderate-risk group is less than 0.001

*P*-value of Low-risk group vs High-risk group is less than 0.001

*P*-value of Moderate-risk group vs High-risk group is less than 0.001

**Fig. 2:**
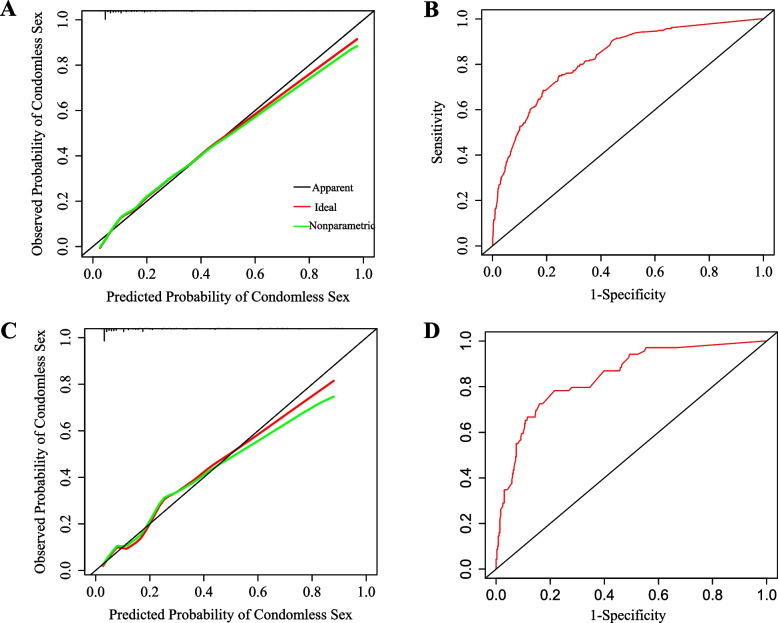
The area under the receiver-operator characteristic curves (AUROC) and the calibration curve of the nomogram for predicting condomless sex. **A** The AUROC of Derivation cohort. **B** The calibration curve of Derivation cohort. **C** The AUROC of Validation cohort. **D** The calibration curve of Validation cohort

In the derivation and validation cohorts, the AUROC of the nomogram prediction model was 0.83 (95% CI, 0.80–0.85) and 0.85 (95%CI: 0.80–0.90), respectively (Fig. 2). The calibration plots showed graphically that the predicted estimate was in good agreement with the ideal value (Fig. 2).

## Discussions

In this cross-sectional survey, the proportion of condomless sex at last intercourse reported in our study (18.2%) was lower than that of the students with similar demographics from other universities in 15 provinces and cities in China (27.4%) [35], and lower than that of the students from the Philippines (83.0%), Indonesia (61.3%), Laos (62.3%), Thailand (58.2%), Singapore (57.8%), Myanmar (48.3%) and Cambodia (42.6%), but higher than that of Malaysia (8.0%) and Vietnam (13.6%) [2]. The different proportions of condomless sex at last intercourse among college students could be attributed to differences in study settings, populations, health education policies, and so on [10, 36]. Efforts were still needed to make to spread knowledge about safe sexual health among college students to facilitate the implementation of the Healthy China 2030.

Our results showed that students who had heterosexual intercourse were less likely to have condomless sex, which we need to explain in detail. Because the whole population in this study had sex in the last year, individuals who had heterosexual intercourse included those who had only heterosexual intercourse and those who had both heterosexual and homosexual intercourse. Students who had not had heterosexual intercourse refer to those who had only had homosexual intercourse. Therefore, it could be concluded from our results that students who had only heterosexual intercourse and those who had both heterosexual and homosexual intercourse were less likely to have condomless sex than those who had only homosexual intercourse, which is similar to what was reported in the study by Cathy Maulsby [37]. This may be because many students still believed that the purpose of condom use was contraception, which increased the likelihood of condomless sex among students who had had homosexual intercourse [37]. It is worth noting that having anal sex was also a risk factor for condomless sex in our study. Given the two factors above, we could further conclude that having had homosexual anal sex was associated with a higher likelihood of having had condomless sex. This finding reminded the universities that it was men who had sex with men who were the focus of the students.

Consistent with existing studies [12–14], our study showed that condom use at first sex was a protective factor for condomless sex. There may be a cognitive link between sexual behavior and condom use at first sex, with students choosing to use condoms at subsequent sex based on the habits of their first sexual experience [12–14]. In addition, condom use at first sex could lead to a reduction in the risk of STIs, and this feedback helped students to use condoms the next time [38]. Previous studies in different populations have suggested that victims of IPV had a higher risk of condomless sex [8, 15]. In addition, our study contributed to the existing literature by adding that having more types of IPV was a risk factor for condomless sex among college students, which may be explained by an accumulated effect of physical, psychological, and sexual victimization. It has been reported that IPV may be caused by the power of oppression [39]. The more types of IPV experienced and the more aspects were oppressed and hurt, the more disadvantaged it was to negotiate safe sex, which was prone to condomless sex [8].

Our findings were in line with several previous studies conducted among college students, which revealed that higher levels of condom use self-efficacy promoted condom use at last intercourse among college students [10, 17]. Condom use self-efficacy has been considered a crucial part of many theories of condom use, including the Theory of Reasoned Action (TRA), Social Cognitive Theory (SCT) and the Health Belief Model (HBM) [28]. In addition, condom use self-efficacy was one of the most important predictors of condom expectancy and actual use [16]. Our results revealed that the higher the positive attitude towards condom use, the higher the likelihood of condom use. This finding was also reported in a systematic review of 96 studies [40]. In other words, if an individual feels that the negative consequences of not using a condom (STIs or unintended pregnancy) are unbearable, or if an individual feels that there are benefits to using a condom, then the individual’s attitude towards condom use is positive [41]. However, it was interesting to note that the norms on condom use were not included in the nomogram in our study, which needs further investigation. The previous studies [40, 41] mentioned that norms on condom use contributed less to the prediction of condom use compared to attitude and self-efficacy, which may partly explain the underlying reasons.

The findings above reminded us to pay more attention to students who have only had homosexual intercourse or anal sex, especially those who have sex with men. In addition, comprehensive sexuality education should be popularized at an early age to promote the use of condoms at first sex so that students can develop the habit. For students who are victims of IPV, professional and effective services could be provided to protect their physical and mental health and reduce their fear of safe sex. Most importantly, it is necessary to establish positive attitudes towards condom use and improve condom use self-efficacy among college students.

Our study developed an effective and convenient tool to calculate the individualized possibility of condomless sex among college students, which showed high accuracy with an AUROC of 0.83 and good discrimination in predicting condomless sex. The nomogram was a visual graph based on a multivariable logistic regression model, where each variable corresponded to a specific point, and the total score of each variable was calculated to quantify the risk of events in the population. This method is effective and convenient, and can convert intangible risks into visual and quantifiable scores. There were various factors associated with condomless sex, and many college students were unable to recognize or identify their risks and therefore did not take any protective measures. We minimized the influence of multicollinearity using LASSO regression, identifying the predictors from multiple perspectives and presenting them in the nomogram. In our nomogram, each college student could calculate a total score based on their actual risk factors for condomless sex and assess their possibility of having condomless sex, which was a quantitative way of acknowledging their risks. Only by understanding their risks can students better guide their practical actions.

Students played an essential role in education, as recipients and practitioners of sexuality education. Each student was responsible for their own health and had to take responsibility for their own consciousness, behavior and consequences [42]. If students were aware of their risks, they could receive individualized education to adapt their behavior, change existing risk factors and reduce the subsequent risks. Our nomogram standardized the scores of the risk factors so that students could recognize the contribution of each factor to the risk of condomless sex, find out the most important risk factors, and receive individualized education on behavior modification and habit change to reduce the influence of the risk factors in the right order.

In addition, our nomogram could be a source of information for individualized education. Only by accurately understanding students’ individualized information can the best personalized teaching strategies be developed for the students. Our nomogram which collected information from students, was used to quantify the individual possibility of condomless sex among college students, which could provide specific recommendations for schools to develop learning plans and training programs. In addition, our study also provided the risk subgroups. For universities and colleges, hierarchical management could be applied to the existing management system to correct the risk factors. For students in the low-risk group, regular education could continue, and for the moderate-risk and high-risk groups, more frequent and intensive education and interventions could be implemented. In the long term, the nomogram could also be used to assess the trend in students’ risks. Universities and colleges could use a series of cross-sectional or cohort studies to assess the risk of condomless sex among freshmen and then implement interventions based on hierarchical management. Continuous assessments could be conducted every 6 months or annually among different subgroups to tailor targeted interventions to each subgroup of students.

Some limitations of this study should be noted. First, the cross-sectional design does not allow conclusions to be drawn about causality [23, 34]. Therefore, prospective studies are needed to verify the predictors of condom use at last sex. Second, the nomogram model was constructed among college students at six universities in Zhuhai City, Guangdong Province, and it needs further external validation among college students in other regions. Although probability proportional to size sampling was used to determine the sample size of each university, in practice there was a gap between the projected proportion and the actual proportion, possibly because the actual number of students drawn from some universities was much larger than the projected number, resulting in a disproportionality and affecting the representation of the sample size. Third, because we relied on self-reported condom use at last sex, we cannot rule out reporting bias, but the anonymity of the questionnaire may reduce the impact of reporting bias.

## Conclusions

Our findings highlighted that students who have only had homosexual sex or anal sex need more attention and that victims of IPV need professional and effective services. To help students use condoms, comprehensive sexuality education should be provided early, and positive attitudes towards condom use and condom use self-efficacy should be improved. In addition, the constructed nomogram prediction model had good discrimination and calibration, and it could predict the individualized risk of condomless sex among college students so that universities and colleges could formulate appropriate individualized measures and sexual health education programs.

### Supplementary Information

**Supplementary Material 1.**

## Data Availability

All relevant data generated during this study will be made available by the corresponding author upon reasonable request.

## References

[1] HIV and AIDS - Basic facts | UNAIDS. https://www.unaids.org/en/frequently-asked-questions-about-hiv-and-aids#how-can-hiv-infection-be-prevented. Accessed 19 Dec 2022.

[2] Yi S, Te V, Pengpid S, Peltzer K (2018). Social and behavioural factors associated with risky sexual behaviours among university students in nine ASEAN countries: a multi-country cross-sectional study. SAHARA J..

[3] Cai C, Tang HL, Chen FF, Li DM, Lv F (2020). Characteristics and trends of newly reported HIV infection in young students in China, 2010-2019. Chin J Epidemiol..

[4] Li G, Jiang Y, Zhang L (2019). HIV upsurge in China’s students. Science..

[5] Wang H, Long L, Cai H, Wu Y, Xu J, Shu C (2015). Contraception and unintended pregnancy among unmarried Female University students: a cross-sectional study from China. PLoS One..

[6] Fonner VA, Kennedy CE, O’Reilly KR, Sweat MD (2014). Systematic assessment of condom use measurement in evaluation of HIV prevention interventions: need for standardization of measures. AIDS Behav..

[7] Espada JP, Morales A, Guillén-Riquelme A, Ballester R, Orgilés M (2015). Predicting condom use in adolescents: a test of three socio-cognitive models using a structural equation modeling approach. BMC Public Health..

[8] Bergmann JN, Stockman JK (2015). How does intimate partner violence affect condom and oral contraceptive use in the United States?. Contraception..

[9] Zhao YL, Kim H, Peltzer J (2017). Relationships among substance use, multiple sexual partners, and Condomless sex. J Sch Nurs..

[10] Elshiekh HF, Hoving C, de Vries H (2020). Exploring determinants of condom use among university students in Sudan. Arch Sex Behav..

[11] Zhou Q, Weizi W, Yi M, Shen Y, Goldsamt L, Alkhatib A (2022). HIV knowledge, sexual practices, condom use and its associated factors among international students in one province of China: a cross-sectional study. BMJ Open..

[12] Shafii T, Stovel K, Davis R, Holmes K (2004). Is condom use habit forming? Condom use at sexual debut and subsequent condom use. Sex Transm Dis..

[13] Shafii T, Stovel K, Holmes K (2007). Association between condom use at sexual debut and subsequent sexual trajectories: a longitudinal study using biomarkers. Am J Public Health..

[14] Guleria S, Thomsen LT, Munk C, Nygård M, Hansen BT, Elfström KM (2019). Contraceptive use at first intercourse is associated with subsequent sexual behaviors. Contraception..

[15] Orchowski LM, Yusufov M, Oesterle D, Bogen KW, Zlotnick C (2020). Intimate partner violence and coerced unprotected sex among young women attending community college. Arch Sex Behav..

[16] Baele J, Dusseldorp E, Maes S (2001). Condom use self-efficacy: effect on intended and actual condom use in adolescents. J Adolescent Health..

[17] Ajayi AI, Ismail KO, Akpan W (2019). Factors associated with consistent condom use: a cross-sectional survey of two Nigerian universities. BMC Public Health..

[18] Iasonos A, Schrag D, Raj GV, Panageas KS (2008). How to build and interpret a nomogram for cancer prognosis. J Clin Oncol..

[19] Lei Z, Li J, Wu D, Xia Y, Wang Q, Si A (2016). Nomogram for Preoperative Estimation of Microvascular Invasion Risk in Hepatitis B Virus–Related Hepatocellular Carcinoma Within the Milan Criteria. JAMA Surg..

[20] Liang W, Liang H, Ou L, Chen B, Chen A, Li C (2020). Development and validation of a clinical risk score to predict the occurrence of critical illness in hospitalized patients with COVID-19. JAMA Intern Med..

[21] Peng R, Liang Z, Chen K, Li L, Qu S, Zhu X (2021). Nomogram based on lactate dehydrogenase-to-albumin ratio (LAR) and platelet-to-lymphocyte ratio (PLR) for predicting survival in nasopharyngeal carcinoma. J Inflamm Res..

[22] Bogani G, Lalli L, Sopracordevole F, Ciavattini A, Ghelardi A, Simoncini T (2022). Development of a nomogram predicting the risk of persistence/recurrence of cervical dysplasia. Vaccines (Basel)..

[23] Ma L, Wang Q, Li X, Shang Y, Zhang N, Wu J (2024). Development of a risk assessment model for cardiac injury in patients newly diagnosed with acute myeloid leukemia based on a multicenter, real-world analysis in China. BMC Cancer..

[24] Qiao Y (2017). Factors influencing health risk behaviors among college students in Zhuhai City, China.

[25] Berhanu A, Mengistu D, Temesgen L, Mulat S, Dirirsa G, Alemu FK (2022). Hand washing practice among public primary school children and associated factors in Harar town, eastern Ethiopia: an institution-based cross-sectional study. Front Public Health..

[26] Guangzhou Center for Disease Control and Prevention. AIDS sentinel surveillance questionnaire and related forms in 2013. http://www.gzcdc.org.cn/download/index/291.html. Accessed 21 Dec 2022.

[27] Dunkle KL, Wong FY, Nehl EJ, Lin L, He N, Huang J (2013). Male-on-male intimate partner violence and sexual risk behaviors among money boys and other men who have sex with men in Shanghai. China Sex Transm Dis..

[28] Basen-Engquist K (1999). Validity of scales measuring the psychosocial determinants of HIV/STD-related risk behavior in adolescents. Health Educ Res..

[29] Rammstedt B, John OP (2007). Measuring personality in one minute or less: a 10-item short version of the big five inventory in English and German. J Res Pers..

[30] Hoyle RH, Stephenson MT, Palmgreen P, Lorch EP, Donohew RL (2002). Reliability and validity of a brief measure of sensation seeking. Personal Individ Differ..

[31] Liao W, Lau JTF, Tsui HY, Gu J, Wang Z (2015). Relationship between sexual compulsivity and sexual risk behaviors among Chinese sexually active males. Arch Sex Behav..

[32] Straus MA (1979). Measuring Intrafamily conflict and violence: the conflict tactics (CT) scales. J Marriage Fam..

[33] Greenwood G, Relf M, Huang B, Pollack L, Canchola J, Catania J (2002). Battering victimization among a probability-based sample of men who have sex with men. Am J Public Health..

[34] Li G, Mei J, You J, Miao J, Song X, Sun W (2019). Sociodemographic characteristics associated with adolescent depression in urban and rural areas of Hubei province: a cross-sectional analysis. BMC Psychiatry..

[35] Ge L, Li D, Li P, Guo W, Cui Y (2017). Population specific sentinel surveillance for HIV infection, syphilis and HCV infection in China, during 2010-2015. Disease Surveillance..

[36] Sun X, Liu X, Shi Y, Wang Y, Wang P, Chang C (2013). Determinants of risky sexual behavior and condom use among college students in China. AIDS Care..

[37] Duby Z, Colvin C (2014). Conceptualizations of heterosexual anal sex and HIV risk in five east African communities. J Sex Res..

[38] Lantos H, Bajos N, Moreau C (2016). Determinants and correlates of preventive behaviors at first sex with a first partner and second partner: analysis of the FECOND study. J Adolesc Health..

[39] Manlove J, Welti K, Karpilow Q (2019). Relationship violence typologies and condom use in young adult dating relationships. Perspect Sex Repro H..

[40] McEachan RRC, Conner M, Taylor NJ, Lawton RJ (2011). Prospective prediction of health-related behaviours with the theory of planned behaviour: a meta-analysis. Health Psychol Rev..

[41] Conner M (2009). Predicting health behaviour: research and practice with social cognition models.

[42] Tan X, Zhang Y, Shao H (2019). Healthy China 2030, a breakthrough for improving health. Glob Health Promot..

