# Supplementary material for: Development of a risk estimation model for condomless sex among college students in Zhuhai, China: a cross-sectional study
Source: BMC Public Health. 2024 Mar 8;24:742. doi: 10.1186/s12889-024-18183-9 (PMC10921646; doi:10.1186/s12889-024-18183-9)
Supplement: Supplementary file 1 — Supplementary Material 1. [file 12889_2024_18183_MOESM1_ESM.docx]

**Supplementary Table 1. Total points in nomogram and the corresponding possibility of condomless sex**

| **Total Points** | **Possibility of condomless sex** |
| --- | --- |
| 30 | 0.05 |
| 65 | 0.10 |
| 104 | 0.20 |
| 130 | 0.30 |
| 151 | 0.40 |
| 170 | 0.50 |
| 190 | 0.60 |
| 211 | 0.70 |
| 237 | 0.80 |
| 275 | 0.90 |
| 311 | 0.95 |
| 390 | 0.99 |

Total points$=48\times Having heterosexual intercourse+22\times types of IPV+26\times Ever having anal sex+79\times Condom use at first sex+7\times\left( 15-Attitudes towards condom use points \right)+8\times(15-Condom use self efficacy points )$

Linear prediction $= 1.6844829+0.3768109\times Having heterosexual intercourse+1.5875592\times types of IPV+1.7474626\times Ever having anal sex+5.0891775\times Condom use at first sex+0.8680907\times Attitudes towards condom use points+0.8379266\times Condom use self efficacy points$

Possibility = 1/ (1+exp (-Linear Prediction)).


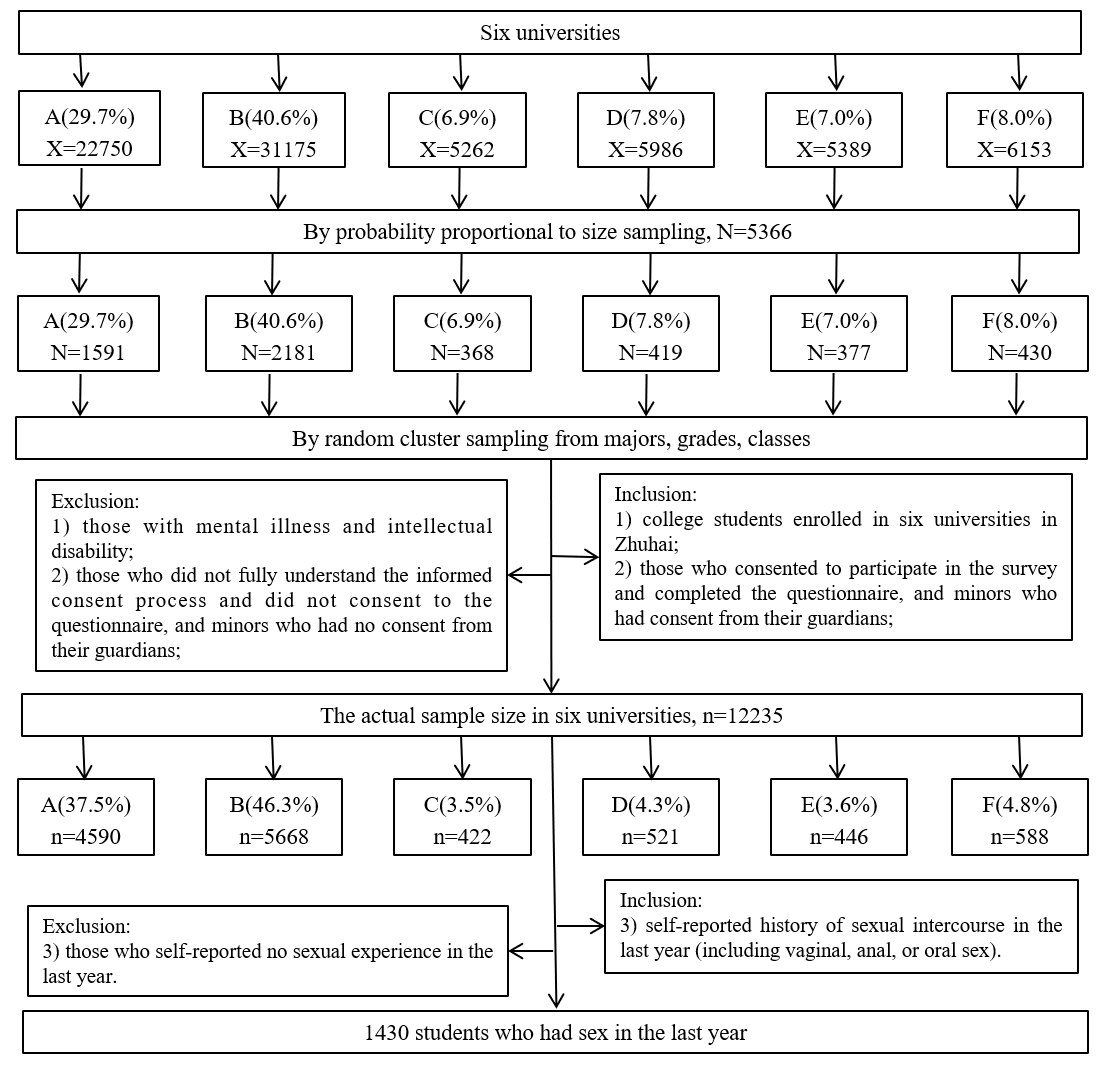


**Supplementary Figure 1. A schematic presentation of sampling procedure for this study.**

**
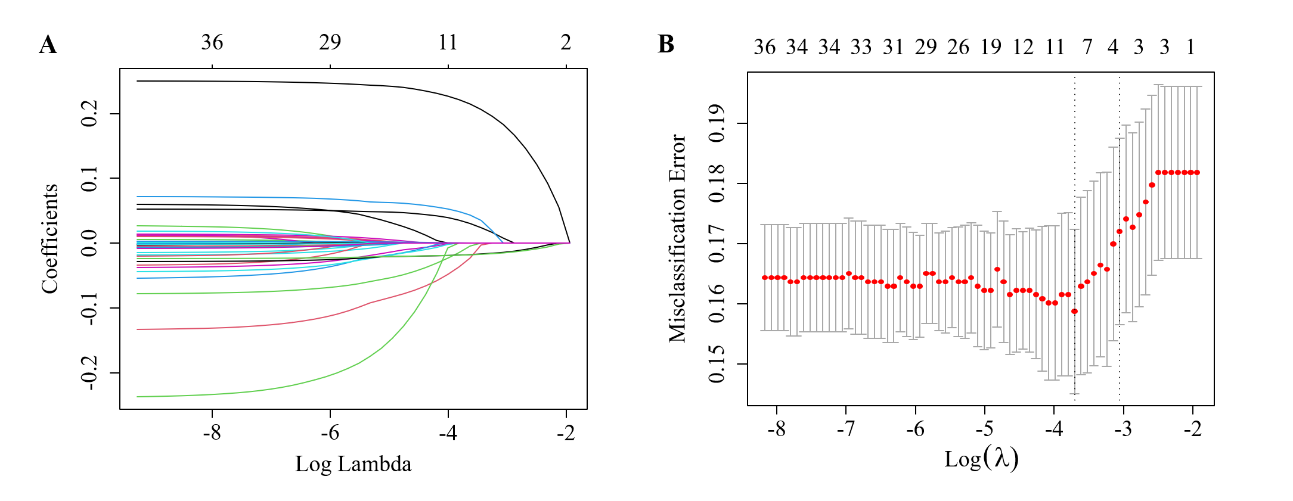
**

**Supplementary Figure 2. Feature selection using the least absolute shrinkage and selection operator (LASSO) logistic regression model**

A: LASSO coefficient profiles of the 36 features among college students who had sex in the last year

B: Tuning parameter (λ) selection in the LASSO model used 10-fold cross-validation via minimum criteria among college students who had sex last year in the last year
